# Toxic Relationships: The Experiences and Effects of Psychopathy in Romantic Relationships

**DOI:** 10.1177/0306624X211049187

**Published:** 2021-10-06

**Authors:** Adelle Forth, Sage Sezlik, Seung Lee, Mary Ritchie, John Logan, Holly Ellingwood

**Affiliations:** 1 Carleton University, Ottawa, ON, Canada; 2 University of Ottawa, ON, Canada; 3 Western University, London, ON, Canada; 4 Public Safety Canada, Ottawa, ON, Canada

**Keywords:** victimization experiences, PTSD, depression, coping strategies, psychopathy, intimate partner violence

## Abstract

Limited research exists on the impact of psychopathy within romantic relationships. We examined mental and physical health consequences reported by intimate partners of individuals with psychopathic traits. Additionally, we explored whether psychopathy severity and coping impacted the severity of posttraumatic stress disorder and depression symptoms. Four hundred fifty-seven former and current intimate partners of individuals with psychopathic traits were recruited from online support groups. Victims reported a variety of abusive experiences and various negative symptomatology involving emotional, biological, behavioral, cognitive, and interpersonal consequences. Psychopathy severity and maladaptive coping were significantly related to increased PTSD and depression, while adaptive coping was only related to decreased depression. Regression analyses revealed that experiencing many forms of victimization predicted increased PTSD and depression symptoms. Examining the specific consequences experienced by intimate partners of individuals with psychopathic traits can aid the development of individualized treatment interventions aimed at symptom mitigation, recovery, and prevention of future victimization.

“Not surprisingly, many psychopaths are criminals, but many others remain out of prison, using their charm and chameleon-like abilities to cut a wide swath through society and leaving a wake of ruined lives behind them.” (Hare, 1993, p. 2).

Psychopathic individuals have a propensity for malevolent and harmful behavior (Blais et al., 2014; Robertson & Knight, 2014; Yang et al., 2010). Despite substantial research on individuals with psychopathic traits, the experience of those victimized by psychopathic individuals has received limited attention. The small number of studies investigating the effects of interacting with individuals with psychopathic characteristics (e.g., Humeny et al., 2021; Kirkman, 2005; Leedom et al., 2012) indicate that these interactions cause considerable physical, sexual, emotional, and/or financial harm. These findings reinforce the view that although the prevalence of psychopathy in the general population is less than 1% (Coid et al., 2009; Neumann & Hare, 2008), the impact on victims’ psychological and physical health is substantial (Boddy, 2014; Kirkman, 2005; Leedom, 2017; Mathieu et al., 2014). In light of the limited number of studies investigating the effects of interacting with individuals who have psychopathic characteristics, the current study was designed to focus on the experiences of victims who self-identified as being intimately involved with a partner with psychopathic traits.

## Overview of Psychopathy

Psychopathy is a syndrome characterized by interpersonal (i.e., grandiose, deceitful), affective (i.e., lack of remorse, shallow emotions), lifestyle (i.e., impulsivity, risk-taking), and social deviance (i.e., poor anger control, criminal activities) features (Hare, 2003). The combination of these traits can have catastrophic effects on the individual who possesses them, as well as those they encounter. Psychopathic individuals tend to act impulsively and are not fazed by the consequences of their actions, so long as those actions lead to immediate self-gratification or personal gain. Further, these individuals lack moral sensibility; they will endorse and engage in immoral behaviors at a greater frequency than non-psychopathic individuals (e.g., Arvan, 2013; Ritchie & Forth, 2016); they simply do not care how their actions will impact others. Although the prevalence of psychopathy in the general population is estimated to be less than 1% (Coid et al., 2009; Neumann & Hare, 2008), the impact on victims’ psychological and physical health is substantial (Boddy, 2014; Kirkman, 2005; Leedom et al., 2012; Mathieu et al., 2014). Psychopathy has been identified as a major risk factor for a multitude of heinous actions, ranging from corporate bullying (e.g., Boddy, 2011) to lethal violence (Fox & DeLisi, 2019). Although much of the research on psychopathy has focused on those with psychopathic traits, the impact on those who survive interactions with these individuals must also be considered.

## Intimate Partner Violence and Psychopathy

Psychopathy is a major risk factor for engaging in intimate partner violence (IPV), which is defined as any violence (physical, sexual, and/or emotional) that occurs within an intimate relationship. In a comprehensive meta-analysis, Robertson et al. (2020) identified psychopathy as one of the strongest predictors of IPV compared to other known risk factors (e.g., aggression, antisocial behavior, alcohol use). Findings were inconsistent about which dimension of psychopathy was most strongly related to IPV perpetration. The one dimension that appears to be most consistently related to IPV is the affective dimension (Cunha et al., 2020; Mager et al., 2014), although additional research across samples and types of IPV is needed to confirm this finding. In general, those with psychopathic traits are more likely to engage in IPV than those without (Grann & Wedin, 2002). As many as 15% to 30% of IPV perpetrators are estimated to meet clinical criteria for psychopathy (Huss & Langhinrichsen-Rohling, 2000).

### Prevalence and impact of IPV

The prevalence of intimate partner violence (IPV) is a global problem, occuring at relatively high rates, especially for women (World Health Organization, 2013). In the United States, approximately 1 in 4 women and 1 in 10 men have experienced physical (women: 21.4%, men: 14.9%) or sexual violence (women: 18.3%, men: 8.2%; Smith et al., 2018). Much higher prevalence rates are found if emotional abuse (e.g., threatened, belittled, or humiliated in front others, insulted or made to feel bad about themselves), financial abuse (e.g., preventing access to family income), or cyber abuse are surveyed (Brem et al., 2019; Sanz-Barbero et al., 2018; Žukauskienė et al., 2021).

Victims in intimate relationships often experience multiple forms of abuse (e.g., Katz et al., 2008). The term polyvictimization has been used to describe this phenomenon (Hamby et al., 2012). Several studies have found that polyvictimization is associated with more negative outcomes (e.g., attachment dysfunction, sexual problems, and negative mental health symptoms) than experiencing a single type of abuse in adolescents and adults (Katz et al., 2008; Ross et al., 2019; Sabrina & Straus, 2008; Turner et al., 2010). In a review of mental health outcomes and IPV, Lagdon et al. (2014) concluded that experiencing multiple forms of abuse increased the severity and incidence of mental health problems. Further, certain characteristics, such as offender-victim relationship, physical injury severity, and type of crime influence victims’ psychological symptoms. Intimate and/or close relationship of victims with perpetrators increases the likelihood of posttraumatic stress disorder (PTSD), anxiety, and depression symptoms compared to acquaintance or stranger encounters, particularly in sexual abuse cases (Gutner et al., 2006; Spencer et al., 2019; Temple et al., 2007), but also for physical assault survivors (Lawyer et al., 2006). Possible explanations for why these effects are larger in more intimate relationships include greater emotional and financial investment (Culbertson & Dehle, 2001; Lawyer et al., 2006) and the increased potential for repeated versus isolated incidents because of easy access (Temple et al., 2007).

Psychological forms of IPV may be the most predictive of negative mental health outcomes compared to physical and sexual forms of abuse (Coker et al., 2002; Norwood & Murphy, 2012). For example, Dutton et al. (1999) examined the three different types of IPV as predictors of PTSD in a sample of court-involved IPV victims. Although univariate analyses showed all forms of IPV to be predictive of PTSD symptoms, a multivariate model indicated that psychological violence explained more variance in PTSD than physical and sexual violence. Studies have also looked at the differential effects of IPV on depressive symptoms. Pico-Alfonso et al. (2006) found that women who were both physically and psychologically abused by their intimate partners had higher rates of depression than those who had been physically abused, strongly suggesting that the psychological form of IPV is not a minor type of violence, but rather a key determinant of mental health outcome.

### Coping and victimization impact

How individuals cope with victimization experiences (i.e., general crime, bullying, and IPV) can affect psychological, physiological, and interpersonal outcomes (Casarez-Levison, 1992). McCann et al. (1988) developed a theoretical model of psychological adaptation in victim responses to trauma, categorizing responses as behavioral, biological, cognitive, emotional, and interpersonal. The model provides insight into individual differences in short- and long-term reactions to traumatic events and provides a framework for exploring the effects of psychopathy.

Many theories of coping describe how a person engages in cognitive and behavioral efforts to manage their victimization experience (Folkman, 2008; Lazarus & Folkman, 1984; Tedeschi & Calhoun, 1996). Generally, coping serves two major functions: (1) dealing with the problem that is causing the distress (problem-focused coping, e.g., obtaining instrumental social support), and (2) regulating or alleviating emotion (emotion-focused coping; e.g., substance abuse, self-distraction). Although the effectiveness of coping strategies is largely dependent on the type of stressor, researchers have argued that problem-focused strategies are adaptive because they provide a sense of control which leads to positive outcomes, such as improved mental and physical health (Billings & Moos, 1981; Lazarus & Folkman, 1984). Conversely, emotion-focused strategies may be maladaptive as no effort is made to change or control the stressful situation, therefore leading to increased distress symptoms (Endler & Parker, 1990; Hooberman et al., 2010; Meyer, 2001). For example, frequent use of emotion-focused strategies by IPV survivors has been associated with heightened symptoms of PTSD (Lilly & Graham-Bermann, 2010). Thus, researchers group coping strategies as adaptive or maladaptive (Kirby et al., 2011; Meyer, 2001; Moore et al., 2011), rather than problem-focused or emotion-focused dimensions. The effects of victimization will vary across individuals, with some individuals experiencing negative outcomes and others being able to effectively cope with these experiences.

### Impact of psychopathy

Psychopathic traits clearly play an important role in the perpetration of IPV. However, the experiences of those victimized by psychopathic individuals remain largely unexplored. While it is possible that the experiences of those victimized by psychopathic individuals do not differ from those victimized by non-psychopathic individuals, the converse is also possible. As psychopathic individuals are thought to account for almost a quarter of IPV perpetrators, our findings will likely overlap with some of the existing IPV outcome research. However, given the determinantal impact that individuals with psychopathic traits can have on others (e.g., Humeny et al., 2021), it is worthwhile to explore the potentially *unique* experiences of their victims as a means of providing better support for recovery. Future research may consider exploring the unique impact that other subtypes of IPV perpetrators may have on their victims.

To date, four studies have examined the experiences and impact of psychopathy within romantic relationships. Leedom et al. (2012) used the published memoirs of 10 women who were in long-term intimate relationships to assess psychopathy in their partners via the Psychopathy Checklist-Revised (PCL-R; Hare, 2003). In addition, they carried out a qualitative analysis of their relationships. All the partners scored high on the PCL-R, with scores ranging from 29 to 40. All the women described being manipulated and deceived throughout their relationship, with many describing abuse toward themselves and their children. Kirkman (2005) identified a variety of harmful behaviors using thematic analysis of interviews with 20 women who were romantically involved with men with psychopathic traits. Eight themes emerged from the qualitative analyses: (1) talking the victim into victimization (100%), (2) lying (100%), (3) financial abuse (75%), (4) emotional abuse (100%), (5) multiple infidelities (100%), (6) isolation and coercion (75%), (7) physical assault (40%), and (8) emotional abuse of children (100%). Brown and Leedom (2008) conducted an online survey of 75 women who reported being intimately involved with a man with psychopathic traits. They found that 95% of the women experienced emotional harm, 71% experienced financial harm, 67% experienced professional harm, and 51% experienced sexual harm. Perhaps the most staggering finding was that none of the women surveyed reported no harm. These survivors reported experiencing anxiety and stress symptoms, depressive symptomatology, dissociation, and problems with interpersonal relationships.

More recently, Humeny et al. (2021) examined the association between psychopathy and intimate partner violence in 475 individuals (89% women) who self-identified as being in an abusive romantic relationship. Victims reported experiencing diverse types of abuse with emotional abuse (99%) being most common, followed by deception (95%), financial abuse (83%), physical abuse (62%), and sexual abuse (59%). Examining the different dimensions of psychopathy, the affective dimension was most strongly related to the length of the relationship, the affective, lifestyle, and antisocial dimensions to the degree of physical injury, and all dimensions were related to frequency and versatility of abuse. Overall, these studies suggest being in a intimate relationship with someone with psychopathic traits causes substantial negative impact.

Women in both Kirkman (2005) and Brown and Leedom (2008) described how at the beginning of the relationship their former partners were highly loving and attentive. Individuals with psychopathic traits are able to detect nonverbal and personality cues of vulnerability (Book et al., 2013, 2021; Ritchie et al., 2018, 2019; Visser et al., 2020; Wheeler et al., 2009), to mimic emotions of fear and remorse (Book et al., 2015; Brazil et al., 2021), and to hide feelings of embarrassment and fear when telling deceptive stories (Porter et al., 2011). These findings suggest that individuals with psychopathic traits may be able to identify potentially vulnerable individuals, obtain their trust, before exploiting and harming them. In addition, by being able to feign remorse, individuals with psychopathic traits may be able to manipulate their partners into staying in the relationship. Murray et al. (2012) also found that individuals with high levels of psychopathic traits did not show cognitive dissonance effects when they lied, whereas those with low levels of psychopathic traits did. The combination of psychopathic traits, deceitful abilities, and lack of cognitive dissonance is particularly toxic to those who become romantically involved with such individuals.

## Purpose of Study

The purpose of the present study was to explore the physical and mental health consequences reported by the intimate partners of individuals with psychopathic traits, with a particular focus on determining whether coping strategies and type and diversity of victimization affect the severity of psychological symptoms. Therefore, we addressed three major research questions:

1. *What are the experiences and effects experienced by the intimate partner victims of individuals with psychopathic traits?* Consistent with past research, we predicted victims would more likely report experiencing emotional as opposed to physical harm and that psychopathy scores in victim’s partners would be associated with experiencing polyvictimization and the degree of physical injury (Brown & Leedom, 2008; Humeny et al., 2021; Kirkman, 2005). We anticipated that intimate partner victims would report a variety of negative symptomatology, via cognitive, behavioral, biological, emotional, and interpersonal consequences.
2. *What is the nature of the relationship between psychopathic traits, coping, and post-truamtic stress and depression of victims?* To answer this question, we examined associations between psychopathy severity, coping, and psychological distress. Based on the available literature (Cunha et al., 2021; Mager et al., 2014; Marshall & Holtzworth-Munroe, 2010; Swogger et al., 2007, 2012), we predicted higher psychopathy scores (i.e., severity) would be related to greater PTSD and depression symptoms for intimate partner victims. Research strongly suggests that the type of coping strategies employed by intimate partner victims can either mitigate or amplify psychological distress symptoms, so the current study sought to examine the association between coping (adaptive and maladaptive) and PTSD and depression symptoms. Previous work suggests that greater use of adaptive coping strategies would be associated with decreased PTSD and depression symptoms, while greater use of maladaptive coping would be related to increased PTSD and depression symptoms (Arias & Pape, 1999; Kocot & Goodman, 2003; Mitchell & Hodson, 1983). We also examined whether the coping strategy used moderated the relationship between psychopathy scores and psychological distress.
3. *What type of abuse (physical, sexual, and emotional) is most predictive of PTSD and depression?* It is important to identify the specific types of abuse that most accurately predict psychological distress in those involved in an intimate relationship with individuals who have psychopathic characteristics. The IPV literature suggests that emotional abuse may be one of the more important predictors of PTSD and depression symptoms, above and beyond the effects found for violent victimization (i.e., physical and sexual abuse; Coker et al., 2002; Norwood & Murphy, 2012; Pico-Alfonso et al., 2006). We also examined if polyvictimization was associated with psychological distress because research has found that experiencing multiple types of abuse is related to more negative outcomes (Lagdon et al., 2014). This analysis was exploratory in nature, as these effects have not previously been investigated for intimate partner victims of individuals with psychopathic traits.

## Method

### Participants

Participants were 457 English-speaking males (*n* = 48, 10.5%) and females (*n* = 409, 89.5% between the ages of 21 and 71 (*M* = 45.13, *SD* = 9.34) who were romantically involved with a partner with moderate to high levels of psychopathic traits before or during their study participation. Most victims reported being no longer involved with their intimate partner (*n* = 358, 79.0%). The extent of involvement ranged from a few months to more than 20 years, with most of the relationships lasting between 2 and 5 years (see Table 1). Participants were involved with male (*n* = 405, 88.8%) and female (*n* = 51, 11.2%) intimate partners. Most victims and partners were Caucasian (victims *n* = 403, 88.4%; victim’s partners *n* = 363, 79.6%). Additional sociodemographic characteristics are provided in Table 1. Most participants came from the LoveFraud website referral source (*n* = 368, 80.7%) with others coming from a variety of other psychopathy-related internet sites (Psychopath-Research.com forum; Aftermath: Surviving Psychopathy; Dr. Robert Hare’s website). Participants reported a variety of motives for participating in the research: simply telling their story, acquiring an understanding of psychopathy and/or victimization, encouraging awareness and educating others, helping others, recovery, and validation of the experiences of others.

**Table 1.** Location, Employment, Education Status, and Relationship Length.

Demographic variable	*n*	Percent
Location (*N* = 457)		
Canada	31	6.8
United States	347	75.9
Europe	48	10.5
Other (i.e., Australia, Asia)	31	6.8
Employment status (*N* = 453)		
Not employed (not looking)	46	10.2
Not employed (looking)	33	7.3
Part-time/seasonal	76	16.7
Full-time	280	61.8
Retired	18	4.0
Education (*N* = 454)		
Elementary school	1	0.2
Secondary school	73	16.1
Community college/technical or trade school	127	28.0
University	153	33.7
Graduate school	100	22.0
Relationship length (*N* = 456)		
<6 months	25	5.5
6–12 months	55	12.1
1–2 years	81	17.8
2–5 years	104	22.8
5–10 years	78	17.1
10–20 years	68	14.9
>20 years	45	9.9

### Measures

#### Sociodemographic and relationship questions

Demographic questions asked about age, country of residence, employment status, occupational background, socioeconomic status, and education level, along with the sex and ethnicity of both the participant and their intimate partner. Participants were also asked to identify their relationship (i.e., significant other vs. ex-significant other), whether they are currently involved, length of the relationship, what types of victimization they experienced (i.e., physical, sexual, emotional, spiritual, financial, deceit, or property theft), degree of physical injury they sustained from the victimization (none, mild/no injury, moderate injury/outpatient treatment, extreme injury/hospitalization) and to rate the impact their partner had on their physical and mental health (rare, mild, moderate, or extreme). A polyvictimization measure was calculated by summing the number of victimization types experienced (data were coded no = 0 and yes = 1), with a maximum score of seven.

#### Self-Report Psychopathy Scale–III

The Self-Report Psychopathy Scale–III (SRP–III; Paulhus et al., 2016) is a 64-item self-report inventory designed to measure psychopathic traits in the general population. On a 5-point Likert scale (1 = *disagree strongly* to 5 = *agree strongly*), people rate the extent to which they agree or disagree about each statement with reference to themselves. With an increasing need to focus on the victims of psychopathic individuals (Viding, 2019), several researchers have begun using modified versions of self-report psychopathy scales to obtain other-ratings (e.g., Humeny et al., 2021; Kirkman, 2005). In the absence of access to the potentially psychopathic individual, the other-rater approach provides the closest estimation of psychopathic traits possible. In the present study, SRP-III statements were modified from first person to third person for participants to rate their partner. A “*don’t know*” (0) option was included. Participants who had encounters with more than one individual with psychopathic traits were asked to choose the most recent.

The SRP-III factor structure consists of four factors, with 16-items per factor. The four factors include: Interpersonal Manipulation (IPM; i.e., “Purposefully flatters people to get them on his or her side”), Callous Affect (CA; i.e., “Have people said he or she is cold-hearted”), Erratic Lifestyle (ELS; i.e., “Enjoys doing wild things”), and Criminal Tendencies (CT; i.e., “Has tricked someone into giving him or her money”). Scores for each subscale range from 16 to 80, and total scores range from 64 to 320. It is important to note that subscale and total scores in the current study may be lower than 64 due to the addition of the “don’t know” (0) rating. The psychometric properties of the SRP–III as a self-report measure have been well-established (Gordts et al., 2017; Mahmut et al., 2011; Neal & Sellbom, 2012; Sandvik et al., 2012; Vitacco et al., 2014).

#### Impact of Event scale–Revised

The Impact of Event Scale–Revised (IES–R; Weiss & Marmar, 1997) is a 22-item self-report scale for assessing situation-specific posttraumatic stress symptomatology, such as intrusion (eight items), avoidance (eight items), and hyperarousal (six items). Respondents rated each item according to the degree of distress it caused since their last contact with the most recent psychopath in their life on a 5-point Likert Scale (0 = *not at all*, 1 = *a little bit*, 2 = *moderately*, 3 = *quite a bit*, 4 = *extremely*). The mean item response to each subscale, ranging from 0 to 4, is calculated, therefore, total possible scores can range from 0 to 12. No diagnostic cut-offs exist for PTSD, but higher scores indicate more symptoms. The IES–R is a widely used self-report measure of posttraumatic symptoms due to its demonstrated reliability and validity (Beck et al., 2008; Creamer et al., 2003; Weiss & Marmar, 1997). Recent findings involving various trauma-exposed populations (e.g., survivors of war, workplace bullying, and flood victims) have demonstrated acceptable to excellent internal consistency (α = .72–.95; Craparo et al., 2013), acceptable convergent validity with other scales (e.g., Dissociative Experiences Scale-II; Craparo et al., 2013), and effective diagnostic utility as a screening tool for PTSD (Morina et al., 2013).

#### Beck Depression Inventory–II

The Beck Depression Inventory–II (BDI–II; Beck et al., 1996) is a self-report scale measuring the existence and severity of depressive symptoms in psychiatric and nonpsychiatric populations. The 21 items on the BDI–II include symptoms such as negative feelings about oneself, physical changes, mood indicators, cognitive difficulties, and reduced pleasure. Participants rated the severity of symptoms during the last point of contact with the most recent psychopath in their life. Each item uses a 4-point scale ranging from 0 to 3 with corresponding symptom descriptors increasing in severity. Total BDI–II scores, calculated by summing the ratings for each item, can range from 0 to 63. However, one item addressing suicidal ideation was removed in the present study. Therefore, total scores range from 0 to 60 and the criteria for minimal (0–10), mild (11–16), moderate (17–25), and severe (29–60) depression were pro-rated. The BDI–II has consistently demonstrated strong psychometric properties across various populations (Beck et al., 1996; Dozois et al., 1998; Steer et al., 1997; Wang & Gorenstein, 2013). Additionally, the BDI-II demonstrates effective diagnostic utility to discriminate between depressed and non-depressed individuals (Park et al., 2020; Wang & Gorenstein, 2013).

#### Open-ended question on effects

Participants were asked the following open-ended question “If you experienced other physical and/or mental health symptoms not possibly mentioned in either of the two scales (IES-R and BDI-II) you just filled out, what were they?”

#### Brief COPE

The Brief COPE (Carver, 1997) is a self-report scale in which 14 coping strategies are measured by two items, on a 4-point Likert scale (1 = *not at all*, 2 = *a little bit*, 3 = *a medium amount*, 4 = *a lot*). The adaptive coping strategy scale consists of the following: Active Coping; Planning; Seeking emotional support; Seeking tangible support: Positive reframing; Acceptance; Turning to religion; Humor. The maladaptive coping strategy scale includes the following: Venting; Denial; Substance use; Behavioral disengagement; Self-distraction; Self-blame. Carver et al. (1993) found adaptive coping subscale (8 coping strategies, 16 items) was linked to a variety of desirable outcomes, and conversely the maladaptive coping subscale (6 coping strategies, 12 items) was linked to undesirable outcomes. The Brief COPE has acceptable to good reliability (internal consistency reported α = .60–.80; Choi et al., 2015; Moore et al., 2011) and significant correlations with relevant constructs such as perceived stress and subjective well-being (see Garcia et al., 2018 for a comprehensive review).

### Procedure

Ethical approval for the research was obtained from a mid-sized Canadian University Psychology Department Ethics Committee. A recruitment announcement was posted on websites dedicated either to the scientific study of psychopathy or support for victims of psychopaths. Interested participants provided informed consent prior to being directed to a secure online data collection site. No compensation was provided for participation.

#### Analytical plan

Descriptive statistics were used to describe the prevalence of abuse experiences, impact of abuse, and level of psychopathic traits in partners, coping, and PTSD and depression symptoms. Content analysis was used to code the responses to the open-ended interview question via Nvivo Software, Version 11 (QSR International Pty Ltd, 2015). For the current study, text-based coding was used, where one- to two-word codes were attached to segments of data (i.e., lines, paragraphs) and then grouped together based on similar words to identify themes. These themes were then quantified in order to determine which physical and mental health consequences were most frequently experienced by victims.

Correlational analyses were used to establish whether psychopathic traits in the victim’s partner were predictive of psychological distress symptoms and to examine the association between coping strategies (adaptive and maladaptive) and psychological distress symptoms. To test whether the victims’ coping strategies moderated the relationship between the severity of psychopathic traits in victims’ partners and psychological distress symptoms, two hierarchical multiple regression analyses were conducted. In each regression, Step 1 used SRP-III total scores, Step 2 added adaptive and maladaptive coping, and Step 3 added the interaction between SRP-III scores and each coping strategy.

To determine whether partner psychopathy scores, physical abuse, sexual abuse, or polyvictimization were most salient in predicting psychological distress, regression analyses using the simultaneous entry method were conducted for both PTSD and depression.

## Results

### What are the Experiences and Physical and Mental Health Effects Experienced by the Intimate Partners of Individuals With Psychopathic Traits?

The mean SRP-III total and subscales of the victim’s partner are presented in Table 3. These scores reflect a very high prevalence of psychopathic traits, with the mean SRP-III total of 213.48 being at the 99.8 percentile for community samples and 78.9 percentile for offender samples (Paulhus et al., 2016). SRP-III total and subscale scores yielded a sufficient range to enable analysis of the association between psychopathic traits and victimization experiences and impact.

Victims reported a substantial range of victimization experiences. For physical violence, participants mostly experienced physical assault (*n* = 231, 50.5%), followed by no physical abuse (*n* = 184, 40.3%), with about one-third having experienced sexual abuse (*n* = 145, 31.7%). For nonphysical harmful acts, emotional abuse (*n* = 448, 98.0%), deception (*n* = 438, 95.8%) and financial abuse (*n* = 369, 80.7%), were commonly reported by victims compared to spiritual (*n* = 266, 58.2%), and property theft (*n* = 181, 39.6%). SRP-III total scores were positively correlated with sexual abuse (*r* = .25, *p* < .001) and physical abuse (*r* = .24, *p* < .001). On average, victims reported experiencing 4.57 (*SD* = 1.42) types of abuse. SRP-III total scores were moderately positively correlated with the polyvictimization measure (*r* = .45, *p* < .001).

When asked to rate the severity of physical injury they experienced, participants (*N* = 457) reported none (*n* = 157, 34.4%), mild injury (*n* = 179, 39.2%), moderate injury (*n* = 92, 20.1%), and extreme (*n =* 29, 6.3%). SRP-III total scores were positively correlated with severity of physical injury (*r* = .24, *p* < .001).

Victimization had a moderate impact on physical health functioning of victims, but substantially affected victims’ mental health according to their ratings in Table 2. SRP-III total scores were positively correlated with physical health (*r* = .22, *p* < .001) and mental health impacts (*r* = .13, *p* < .01).

**Table 2.** Impact of Victimization on Physical and Mental Health of Victims.

Degree of impact	Physical health (*N* = 433)		Mental health (*N* = 451)	
	*n*	Percent	*n*	Percent
Rare	70	15.3	2	0.4
Mild	90	19.7	7	1.5
Moderate	163	35.7	79	17.3
Extreme	120	26.3	363	79.4

Physical and mental consequences of being an intimate partner with a psychopathic individual were analyzed both quantitatively and qualitatively. First, victims’ ratings of PTSD and depression symptoms since the last contact with their intimate partner was obtained (see Table 3 for descriptive statistics). Victims rated themselves in the moderate range on PTSD symptoms (with 83.8% scoring above 1.5 on the IES-R, which signifies the likely presence of PTSD according to Creamer et al., 2003) and rated themselves as more affected by intrusive and hyperarousal symptoms than avoidance. Victims’ self-ratings of depression placed a substantial number of them into the moderate range for depression (51.4% scored moderate or higher with 22.5% scoring as extreme). Second, answers to the open-ended interview question on distress were organized using the McCann et al. (1988) model. The open-ended question was answered by 48% of the sample (*n* = 221). This lower response rate was likely due to the question wording that asked participants to describe symptoms not covered in the IES-R and BDI-II. Victims reported many negative direct and indirect effects of victimization (Table 4). Most victims had more than one theme that emerged from their responses.

**Table 3.** Descriptive Statistics and Internal Consistencies for SRP-III Total and Factor Scores, IES-R Total and Subscales, BDI-II Total, and the Brief COPE Adaptive and Maladaptive Scores.

Scales	Range	Mean	*SD*	α
SRP-III				
Interpersonal manipulation	30–79	62.04	9.35	.68
Callous affect	21–77	52.72	9.71	.61
Erratic lifestyle	23–77	56.29	9.32	.62
Criminal tendencies	11–75	42.44	11.78	.67
Total	135–296	213.48	29.89	.83
IES-R				
Avoidance	0.13–4	2.07	0.79	.74
Intrusion	0.25–4	2.93	0.86	.88
Hyperarousal	0–4	2.88	0.93	.82
Total	0.50–12	7.88	2.17	.90
BDI-II				
Total	0–60	21.34	13.04	.94
Brief COPE				
Adaptive	20–64	45.07	9.11	.86
Maladaptive	13–40	26.01	5.29	.64

*Note.* Sample size ranged from 368 to 457. SRP-III = Self-Report Psychopathy Scale (Paulhus et al., 2016); IES-R = Impact of Event Scale (Weiss & Marmar, 1997); BDI-II = Beck Depression Inventory (Beck et al., 1996); Brief COPE (Carver, 1997).

**Table 4.** Themes of Victimization Effects.

Theme	Frequency *n*	Example
Emotional consequences	247	“I was really terrified and anxious because he threatened me so much during and following the divorce. . ..”
Biological consequences	126	“I developed high BP, have migraines, the beginnings of heart disease, just feel that my physical health will never be the same.”
Behavioral changes	109	“There were times when I would not sleep for two complete days. . .”
Cognitive changes	68	“Difficulty concentrating, obsessing, thinking about him constantly, wondering why?”
Interpersonal consequences	38	“I have a profound distrust of myself. . .”

*Note.* Victims identified multiple different themes in their answers.

#### Qualitative analysis of open-ended question responses

##### Emotional consequences

The most prevalent consequences reported by victims were psychological/emotional difficulties. Victims reported feelings along the dimensions of anger (i.e., irritability, frustration) and hatred (i.e., of self, misogyny). (The responses reported below are in verbatim form, uncorrected for grammar, spelling, or punctuation.) Typical responses included, “For a while after the relationship, I was angry at having been deceived on such a deep level.” “When it all happened in the end I was having anger and rage.” and “When he stormed out. . ., I felt very strong feelings of ‘how dare he do this to me’ who does he think he is !! I was so ANGRY.”

Feelings of anxiety, fear, panic, and paranoia were common. Victims also often mention being diagnosed with PTSD and having obsessive symptoms: “I feel scared when I am out, I am afraid of bumping into him. I fear he is still going to ruin my life because I got away from him.”; “. . . I just was tired, and obsessed with trying to understand it. . . and there is no understanding of it.. . .”; “I was very nervous most of the day and into the night, having trouble sleeping. I never had such a prolonged feeling of panic or worry like this, ever!”; “I am experiencing a lot of anxiety. The feeling of not having control over my life. I am upset that I let someone in who could affect me so negatively. Panic attacks when I remember one of his big lies.”; and “I lived on high alert trying to protect my children every moment of every day for 19 months. The courts were useless. The police? Useless. Locked doors and windows? Useless.”

Although victims did complete the BDI-II, many of their open-ended responses described depressive symptoms, including anhedonia, suicidal ideation and attempts, hopelessness, helplessness, and low sense of self-worth. Typical responses included: “I hit rock bottom in my life. It was the most painful experience I have ever endured. I for the first time thought of suicide as life was too painful to deal with.”; I became depressed and suicidal. It took me several years to work myself out of it.”; “I have been diagnosed with severe depression. During the last 3 years with this man, I have attempted suicide four times.” and

The lack of sleep, toward the end of the relationship, led to paranoia (also because of his vague and increasingly cruel behavior. Like I was a distasteful chore he had to deal with.). . .. I began feeling inadequate at everything in my life. These feelings reached a peak at which I began feeling so desperate that I contemplated suicide daily.

Some victims described feelings of guilt or shame for staying in the relationship for so long and exposing their family to this individual:

I feel a lot of guilt over what has happened to me because this psychopath stole from my family and I continued to see him hoping he would right his wrong. I didn’t come to the defense as a mother should have when things were happening..I always believed he would have good consious to make it right by them, which of course he didn’t. I feel like my instincts were talking but he schmoozed them and took over every rational thought I had within me.

Several victims mentioned feelings of denial, grief, and disappointment. For example, “A constant aggravation and disappointment in myself for getting involved and then staying involved w/ him for as long as I did. (for letting him talk me into things I would’ve never done).” and

. . .I went back into denial, is the best I can categorize it. I didn’t deal with what it all REALLY meant. I was like a walking zombie, trying to stay on my feet but out of the blue it was like something would kick me in the gut and I would find myself in a pool of tears and not know why. It took me 9 months after the break up before I faced the reality that the whole thing had been a lie, realized the full extent of what it all meant. Realized what he really is. Denial was easier……………maybe not healthier, but easier.

##### Biological consequences

Victims reported a wide range of biological effects, with the most common being somatic problems such as gastrointestinal problems, ulcers, and headaches. One victim said “I had a substantial amount of weight loss. I would gag when I would try to eat. Initially my hair did not grow. My hair fell out and is coming in gray. My nails would not grow. I would be completely dehydrated. I developed a bleeding ulcer. I would not eat sometimes for days.”, while another reported, “Headaches and blinding shooting pains behind eyes backache teeth grinding hand wringing/fiddling aching neck and shoulders pins in needles in fingers staring into space,” and yet another wrote, “Acid reflux, chest pains, feeling sick to my stomach every single day, headaches, dizziness, depressed, extreme anxiety.”

Other common biological consequence were heart and respiratory problems (i.e., angina, asthma, bronchitis). One participant described “I am now on blood pressure meds and experience angina allot.” while another reported “Heart palpitations, shortness of breath, tired all the time.”

A range of endocrine and urologic diseases (i.e., diabetes, hypothyroidism) and autoimmune diseases (i.e., rheumatoid arthritis) were reported. Responses in this category included: “I now believe that my arthritis, colon issues, etc. are directly related to the stresses of living for 14 years with a psychopath. The amount of personal energies required to handle getting through day to day life with him seemed to have sucked health from me personally.” and “My thyroid went wacky. I now have hypothyroidism. My TSH level were surprising to my doctor at 16.4, I am on medication.”

Less common disorders of the central or peripheral nervous systems (i.e., multiple sclerosis, trigeminal neuralgia, sleep apnea) were also reported. For example, “I was diagnosed with Multiple Sclerosis also during that time-the stress caused the MS symptoms to manifest in extremes-fatigue, loss of vision, weakness, and temporary paralysis of the left leg.” and “I was diagnosed with fibromyalgia and Trigeminal Neuralgia, high blood pressure, and depression.”

Some victims mentioned having reproductive problems (i.e., sexually transmitted diseases, fertility problems). For example, “I experienced menstruation symptoms which led me to get extra special medical help. I also experienced infertility problems.” and “He promised me that he had no diseases; I had one experience with him, not using a condom. I now have herpes menstrual is.” and

I was pregnant with twins at 13 wks when the psychopath insisted that I help him erect a storage shed in our backyard during a cold windy night. I told him I was tired and not feeling well, but as usual he had to have his way at all costs, so I helped him and then miscarried the next day.

Finally, some victims described the physical injuries they have received. Responses here included: “My ribs ache at times where he broke them. Lots of other things just cant write them. sorry”, and “My back was broken when i confronted her about an STD. My eye was blacked when I didn’t approve of her meeting on of her druggie friends”

##### Behavioral changes

Victims reported numerous behavioral changes, including changes to sleeping and eating (i.e., insomnia), neglect of self-care (i.e., substance abuse, smoking), and changes in social activities/or interactions. Responses in this theme include the following: “When I was in the ‘eye of the storm’, I had MAJOR problems getting to sleep, and staying asleep, was very irritable, couldn’t concentrate, wasn’t my usual self with colleagues and friends who didn’t know what was going on but sensed there was something a miss with me.”, “self-injury (cutting) panic attacks drug abuse (cocaine, benzo) drink too much” and “Physically, I just do not take care at all. I neglected myself a lot.” Many victims mentioned they reduced social activities such as “I was dealing with nearly constant depression for a long time after I left this woman. I would not venture out in public for too long, instead preferring to stay indoors and alone. I do not socialize anywhere as much as I once used to,” or “I stayed home more frequently, and dropped out of social groups, turned down invitations for all social events.”

##### Cognitive changes

Given the prevalence of PTSD symptoms it is not surprising that many victims reported experiencing intrusion (i.e., flashbacks) and dissociation (i.e., rumination), concentration difficulties (i.e., loss of focus), and memory difficulties. Representative responses include: “I have frequent flashbacks, and am in a near-constant state of nausea.”, “I suffered an acute sense of hypervigilence after being with the sociopath. It lasted nearly two months. I was very nervous most of the day and into the night, having trouble sleeping. I never had such a prolonged feeling of panic or worry like this, ever!”, “ and “I was unable to concentrate and nearly lost my job because my boss noticed I was forgetting things and making mistakes.”

##### Interpersonal relationships

Involvement with an intimate partner with psychopathic traits also had devastating effects on victims’ interpersonal relationships. Many victims developed a loss of trust in others, as illustrated by descriptions of questioning people’s motives, checking out their backgrounds, withholding personal information, and fear of betrayal or abandonment. Victims perceived their own judgments of others as faulty because of their relationship with their partner with high levels of psychopathy. For example, “I used to be a very trusting person. Now I am extremely cautious, especially with men.”, “This has shaken my faith. I reported what happened and I do not think I was believed. Now I don’t trust the others in my religious organization. . .. I feel I am no longer valued by others in my religion, and I used to feel and know I was very valued. It’s hard to believe in myself when they are all believing him and invalidating me. . .The thing that gave me the most meaning in my life has been damaged and may be beyond repair.” and “. . .people at all, and sadly, even some of the people closest to me I have questioned my trust for them too. I have great difficulty telling people about this because of the humiliation involved.” In addition, victims described reported feelings of loss and loneliness: “I feel empty and lonely, all the time. Like I’ve lost everything good in my life because I’m an idiot and fell in love with a psychopath. I have little hope for being able to find someone, or anyone who will ever understand me or want to date me.”

Victims reported interacting differently with people as a result of their victimization. Comments in this theme included: ““I have not been outside my home for over 4 months, I become irritable and try to find excuses when friends or family pressure me into going out somewhere.. I want to move on with my life, but don’t feel that I am ever going to be safe or free from his harassment. . .”, “I stayed home more frequently, and dropped out of social groups, turned down invitations for all social events.”; “I have avoided going to many of the places I once frequented, and I avoid friends who unwittingly participated in the shaming she put me through; they were conned, too, but some of them still don’t understand that.”; and

I do not feel ‘human’. I desire closeness with another but can’t imagine being close to anyone. Can’t imagine having sex with someone or even what sex is. I feel worthless and undesirable. I currently have no male friends or men in my life. I have lost contact with almost everyone the last month or so since I feel I am finally done.

### What Is the Nature of the Relationship Between Psychopathic Traits, Coping, and Psychological Distress of Victims?

Bivariate correlations between SRP-III scores, Brief COPE, IES-R, BDI-II scores were calculated to determine whether psychopathy was associated with coping tactics, PTSD and depression (see Table 5). Higher total psychopathy scores were expected to be associated with higher PTSD and depression symptoms.

**Table 5.** Bivariate Correlations between SRP-III Total and Factors, PTSD Symptoms, Depression Symptoms, and Coping Strategies.

Scale	1	2	3	4	5	6	7	8	9
1. SRP-III Total	—								
2. SRP-III IPM	.74***	—							
3. SRP-III CA	.72***	.49***	—						
4. SRP-III ELS	.73***	.39***	.32***	—					
5. SRP-III CT	.78***	.37***	.36***	.49***	—				
6. IES-R Total	.26***	.25***	.26**	.18**	.12*	—			
7. BDI-II Total	.16**	.15**	.11*	.14**	.09	.54***	—		
8. Brief COPE adaptive	−.06	−.05	−.11*	−.03	−.003	.04	−.32***	—	
9. Brief COPE maladaptive	.10*	.09	.09	.05	.07	.45***	.52***	.00	—

*Note.* Sample sizes ranged from 366 to 457. SRP-III = Self-Report Psychopathy Scale (Paulhus et al., 2016); IPM = Interpersonal Manipulation; CA = Callous Affect; ELS = Erratic Life Style; CT = Criminal Tendencies; IES-R = Impact of Event Scale (Weiss & Marmar, 1997); BDI-II = Beck Depression Inventory (Beck et al., 1996).

* *p <* .05. **p < .01. ***p < .001 (two-tailed).

Significant positive correlations were found between total psychopathy scores and PTSD total scores (see Table 5). Findings were consistent across psychopathy factors, although the Interpersonal Manipulation and Callous Affect were more strongly associated with PTSD symptoms. A small positive correlation was identified between total psychopathy scores and depression (see Table 5). Similarly, the psychopathy factor scores were all positively related to depressive symptomatology.

Bivariate correlations between Brief COPE, IES-R, and BDI-II scores were calculated to determine whether coping was associated with psychological distress (see Table 5). Higher total adaptive coping scores were expected to be associated with lower PTSD and depression symptoms. Inversely, higher maladaptive coping scores were expected to correlate with higher PTSD and depression symptoms. No significant correlations were found between adaptive coping and PTSD total or subscale scores. However, moderate correlations were identified between maladaptive coping and PTSD total scores (*r* = .45, *p* < .01). Moderate to strong correlations were also found between adaptive and maladaptive coping and depression scores (*r* = −.32, *p* <.01; *r* = .52, *p* < .01, respectively). Notably, the direction of the association differed across adaptive and maladaptive coping strategies, where adaptive coping scores were negatively related to depression and maladaptive coping scores were positively correlated with depression.

To test whether the victims’ coping strategies moderated the relationship between the severity of psychopathic traits in victims’ partners and psychological distress symptoms, hierarchical multiple regression analyses were conducted (see Table 6). In the first step, only SPR-III total scores were included. This variable accounted for a significant but small amount of variance in PTSD and depression symptoms, *R*^2^ = .063, *F*(1, 364) = 24.64, *p* < .001; *R*^2^ = .021, *F*(1, 366) = 7.82, p = .005. Specifically, increased psychopathic traits in the victims’ partner were significantly associated with a higher level of psychological distress in victims.

**Table 6.** Hierarchical Multiple Regression Analyzes for Psychopathy and Coping Strategies Predicting PTSD and Depression.

	*B*	*SE* (*B*)	95% CI	β	*p*
*PTSD*					
STEP 1					
Intercept	7.90	0.11	[7.68, 8.12]		<.001
SRP-III	0.54	0.11	[0.32, 0.76]	.25	<.001
STEP 2					
Intercept	7.90	0.10	[7.70, 8.10]		<.001
SRP-III	0.45	0.10	[0.25, 0.65]	.21	<.001
COPE-Adaptive	0.13	0.10	[–0.07, 0.33]	.06	.20
COPE-Maladaptive	0.94	0.10	[0.74, 1.14]	.43	<.001
STEP 3					
Intercept	7.90	0.10	[7.70, 8.10]		<.001
SRP-III	0.43	0.10	[0.23, 0.63]	.20	<.001
COPE-Adaptive	0.14	0.10	[–0.06, 0.34]	.07	.15
COPE-Maladaptive	0.94	0.10	[0.74, 1.14]	.43	<.001
SRP–III × Adaptive	−0.12	0.10	[–0.32, 0.08]	−.06	.25
SRP–III × Maladaptive	−0.04	0.11	[–0.24, 0.16]	−.02	.69
*Depression*					
STEP 1					
Intercept	21.63	0.67	[–1.31, 17.79]		<.001
SRP-III	1.88	0.67	[0.02, 0.10]	.15	.005
STEP 2					
Intercept	21.68	0.54	[–7.84, 13.06]		<.001
SRP-III	0.95	0.54	[–0.001, 0.07]	.07	.08
COPE-Adaptive	−4.13	0.54	[–0.57, –0.33]	−.32	<.001
COPE-Maladaptive	6.66	0.54	[1.06, 1.46]	.51	<.001
STEP 3					
Intercept	21.59	0.54	[–7.84, 13.06]		<.001
SRP-III	0.87	0.55	[–0.001, 0.07]	.07	.11
COPE-Adaptive	−4.04	0.55	[–0.57, –0.33]	−.31	<.001
COPE-Maladaptive	6.59	0.54	[1.06, 1.46]	.51	<.001
SRP-III × Adaptive	−0.40	0.55	[–0.32, 0.08]	−.03	.47
SRP-III × Maladaptive	0.69	0.58	[–0.24, 0.16]	.05	.24

*Note.* CI = confidence interval for *B*. SRP-III = Self-Report Psychopathy Scale (Paulhus et al., 2016). To avoid potentially problematic high multicollinearity with the interaction term, the variables were standardized, and an interaction term between psychopath severity and coping strategies (adaptive and maladaptive) was created.

In the second step, two variables regarding the types of coping strategies (adaptive and maladaptive) were added to the regression model, which accounted for a significant proportion of the variable in PTSD, Δ*R*^2^ = .188, Δ*F*(2, 362) = 45.50, *p* < .001; however, only the maladaptive coping level was significantly and positively associated with PTSD. Both adaptive and maladaptive levels were associated with depression symptoms of victims, Δ*R*^2^ = .359, Δ*F*(2, 364) = 105.31, *p* < .001. After controlling for these coping variables, SRP-III total scores were no longer associated with depression symptoms of victims. Next, the interaction term between psychopathy scores and coping strategies was added to the regression model; it did not account for a significant proportion of the variance in PTSD, Δ*R*^2^ = .003, Δ*F*(2, 360) = 0.709, *p* = .493, or depression, Δ*R*^2^ = .004, Δ*F*(2, 362) = 1.079, *p* = .341. In other words, coping skills of victims did not moderate the relationship between the severity of psychopathic traits and psychological distress.

### What Type of Abuse (Physical, Sexual, or Emotional) Is Most Predictive of PTSD and Depression?

We could not determine if emotional abuse was more strongly related to psychological distress than other types of abuse because of a ceiling effect for emotional abuse (i.e., virtually all participants reported emotional abuse, resulting in a lack of variability for emotional abuse). The abuse variables were coded dichotomously as not experienced or experienced.

Four predictor variables were included in the regression analyzes: (1) psychopathy scores, (2) physical abuse, (3) sexual abuse, and (4) polyvictimization.

To determine which factors were most salient in predicting psychological distress, regression analyses using the simultaneous entry method were conducted for both PTSD and depression (see Table 7). Overall, the regression model for predicting PTSD was significant, *F* (4, 380) = 9.09, *p* < .001, *R*^2^ = .09. Specifically, higher psychopathic traits in victims’ partners, *β* = .19, *t*(379) = 3.46, *p* = .001, and more types of abuse experiences (polyvictimization), *β* = .19, *t*(379) = 2.45, *p* = .015, significantly predicted higher PTSD symptoms of victims; however, experiencing physical or sexual abuse was not significantly associated with PTSD symptoms.

**Table 7.** Multiple Linear Regression Analyses for Psychopath Severity, Types of Abuse, and Polyvictimization Predicting PTSD and Depression.

	*B*	*SE* (*B*)	95% CI	β	*p*
PTSD					
Intercept	3.70	0.794	[2.14, 5.26]		<.001
SRP–III	0.01	0.004	[0.002, 0.02]	.19	.001
Physical abuse	−0.32	0.28	[–0.87, 0.23]	−.07	.26
Sexual abuse	−0.01	0.28	[–0.56, 0.54]	−.001	.98
Polyvictimization	0.30	0.12	[0.06, 0.54]	.19	.02
Depression					
Intercept	5.24	4.92	[–4.40, 14.88]		.29
SRP–III	0.04	0.03	[–0.02, 0.10]	.10	.10
Physical abuse	−2.47	1.75	[–5.90, 0.96]	−.10	.16
Sexual abuse	−1.24	1.71	[–4.59, 2.11]	−.05	.47
Polyvictimization	1.94	0.75	[0.47, 3.41]	.21	.01

*Note*. CI = confidence interval for *B*. SRP-III = Self-Report Psychopathy Scale (Paulhus et al., 2016).

The regression model for predicting depression was also significant, *F* (4, 374) = 4.316 *p* = .003, *R*^2^ = .04. However, the variable measuring the number of different types of abuse experiences (polyvictimization) was the only predictor for the depression symptoms after controlling for other variables, where the higher number of different types of abuse experiences positively predicted depression symptoms, *β* = .21, *t*(373) = 2.60, *p* = .010.

## Discussion

To address the limited knowledge about victims of community-based individuals with psychopathic traits, the current study explored psychological consequences, distress predictors, and the relationships between psychopathy severity, coping, and distress. Our results strongly suggest that although intimate partner victims of psychopaths experience a variety of physical and mental health consequences, the severity of these symptoms is indeed affected by other factors (i.e., coping strategies, the level of psychopathic characteristics present, type of victimization).

Victims reported experiencing psychological and physiological effects comparable to the symptoms reported by victims of general crime, bullying, workplace bullying, and intimate partner violence (Bowling & Beehr, 2006; Freedy et al., 1994; Hawker & Boulton, 2000; Johansen et al., 2006; Nolfe et al., 2010; Purcell et al., 2005). These consequences were diverse and included emotional consequences, biological effects, behavioral difficulties, and interpersonal difficulties. These findings are consistent with past literature and provide evidence to support the hypothesis that intimate partner victims of psychopaths would report a variety of deleterious physical and mental health consequences, particularly in the form of psychological distress (i.e., PTSD and depression). Notably, most of the victims reported that their intimate involvement with a partner with high levels of psychopathy had resulted in a moderate impact on their physical health and an extreme impact on their mental health. Future research will need to include a comparison sample of individuals who have experienced intimate partner violence by individuals with low levels of psychopathic traits to determine how unique these experiences are.

The current study examined whether psychopathy was related to psychological distress factors commonly experienced by survivors of violent/nonviolent victimization, namely PTSD and depression symptoms. Based on the extant literature, we expected that higher psychopathy scores would be associated with increased PTSD and depression on the part of victims (Baughman et al., 2012; Boddy, 2011; Brown & Leedom, 2008; Kirkman, 2005; Mager et al., 2014; Porter et al., 2003; Ragatz et al., 2011; Swogger et al., 2007). As predicted, higher total and factor psychopathy scores were associated with increased PTSD and depression symptoms. Despite these significant findings, the association between psychopathy and these psychological distress factors was weak. Psychopathy scores accounted for only 6% of variance in PTSD symptoms and 2% of variance in depression symptoms. A possible reason for this result is that a large proportion of victims reported no longer being involved with the psychopath, potentially providing sufficient time to recover from the victimization experiences (Culbertson & Dehle, 2001; Faisal-Cury et al., 2013; Temple et al., 2007). Additionally, other factors (i.e., social support) may have mitigated the relationship between psychopathy and the severity of psychological distress symptoms reported by intimate partners. Finally, and perhaps most importantly for this sample, a ceiling effect may have limited the effect of psychopathy because the psychopathy scores were generally high (mean psychopathy scores corresponded to the 99.8^th^ percentile in community samples and the 78.9th percentile in offender samples, Paulhus et al., 2016), with limited variability. As noted above, future research should include a comparison sample of victims whose intimate partner scored low on psychopathy to help clarify the importance of psychopathy in predicting negative consequences.

In partial support of our hypotheses, increased adaptive coping was associated with decreased depression symptoms, whereas this relationship was not observed for PTSD. Previous research (Kocot & Goodman, 2003; Schnider et al., 2007; Wong et al., 2016) typically examined the specific (i.e., problem-, emotion or avoidance- focused strategies) rather than broad dimensions (i.e., adaptive and maladaptive) of coping, which could offer a possible explanation for the unexpected findings. Specifically, the current study included certain items within the adaptive scale (i.e., humor; “making jokes about it”) that when relied on too heavily or when employed in a maladaptive self-focused fashion (i.e., self-deprecating humor) can lead to detrimental outcomes (e.g., increased anxiety, PTSD symptoms; Kuiper et al., 2004). Conversely, certain items included within the maladaptive coping scale, such as venting one’s emotional distress, have been viewed as an adaptive coping strategy by other researchers (Folkman & Lazarus, 1985). Moreover, despite overlap, differences in the etiology and symptomatic presentation for PTSD and depression (anxiety vs. mood disorder) may partially explain these findings. The lack of significant effects may have also been due to a lack of control for social support resources, victimization severity, prior victimization, or symptom severity. While the current study only examined the relationship between adaptive coping and psychological distress symptoms (i.e., PTSD and depression), previous researchers (i.e., Meyer, 2001) controlled for factors like prior distress symptom severity. Additionally, previous researchers have suggested that problem-focused (i.e., adaptive) coping strategies are more appropriate for stressful or traumatic situations that are considered controllable, whereas emotion-focused (i.e., maladaptive) coping strategies work best for events considered uncontrollable. This offers a possible explanation, as many victims of intimate partner victimization consider their abusive experiences to be uncontrollable. Similarly, intimate partners may anticipate that adaptive coping strategies (i.e., “taking action to try and make the situation better”) could lead to negative outcomes such as increased physical injury or financial repercussions, which would in turn create greater symptoms of anxiety or PTSD.

As predicted, we found that increased maladaptive coping to be associated with increased PTSD and depression symptoms (Arias & Pape, 1999; Mitchell & Hodson, 1983). Despite the debate about whether certain maladaptive items can be considered adaptive in certain contexts and at certain levels (i.e., venting), the current study provides support for the overall maladaptive nature of these coping behaviors, consistent with the extant literature (Endler & Parker, 1990; Meyer, 2001), and supports the hypothesis that intimate partner victims of individuals with psychopathic traits employing maladaptive coping strategies would experience greater psychological distress, by way of PTSD and depression symptoms.

In addition, the current study sought to explore what type of victimization (i.e., emotional, sexual, and physical) was most predictive of psychological distress (i.e., PTSD and depression). We wanted to explore if psychological or emotional abuse would be a significant and unique predictor of psychological distress, above and beyond the effects of sexual and physical victimization (Coker et al., 2002; Norwood & Murphy, 2012). Given the high rates of emotional abuse endorsed by our participants (98%) we were not able to address this prediction. We found that polyvictimization (experiencing multiple different types of victimization) was identified as the only significant predictor of PTSD and depression symptoms in the regression analyses. While some research has pointed to the unique effects of psychological abuse (e.g., Norwood & Murphy, 2012), burgeoning evidence supports this polyvictimization finding. For instance, in the context of dating partner abuse, polyvictimization was found to be the only significant predictor of PTSD and depression symptoms for women (Sabrina & Straus, 2008). Similar results have also been found in the family violence literature, with multiple exposures to abuse or life-course polyvictimization (childhood maltreatment and IPV) resulting in greater risks of experiencing depression and PTSD (Armour & Sleath, 2014; Cavanaugh et al., 2012; Chan et al., 2021; Finkelhor et al., 2007). Of note, the current study used categorical variables in which the victims indicated whether they had experienced this specific type of victimization or not. Future analyses should explore whether the severity of psychological distress symptoms (i.e., PTSD and depression) differs across severity levels of victimization (mild, moderate, extreme), and whether other factors like social support moderate this relationship.

Although the present study obtained several results that increased our understanding of the effects of victimization by intimate partners with high levels of psychopathy, several factors limit its conclusions. First, participants were asked to rate the psychopathic traits of their intimate partners, which could have led to unintentional inaccurate ratings for items in which they lacked familiarity (i.e., “broken into a car or building to steal or vandalize. . .”) or their own biases. To ensure the safety and anonymity of the victims participating in this form of research, current or former partners are not typically contacted for an assessment of psychopathy. However, Brieman and Kosson (2018) examined the association between incarcerated men’s self-reported SRP-III score, their partner’s (i.e., the wives/girlfriends of the men) ratings of the men using the SRP-III (i.e., SRP score obtained by other-rating), and the men’s score on the Psychopathy Checklist-Revised (PCL-R; Hare, 2003). Supporting the accuracy of other-ratings, partner’s SRP-III rating of their partner were more strongly correlated (*r* = .55) with their male partners’ PCL-R scores than their male partner’s self-report (*r* = .27). This pattern of results demonstrates that other-ratings can be an accurate measure of psychopathy.

Another source of sample bias stemmed from the characteristics of the intimate partners themselves. The sample was largely recruited from online support groups and therefore these intimate partner victims were likely coping better than those who were not involved in such support groups. Specifically, because they were already members of online support groups and were receiving coping and social support resources, the severity of their psychological distress symptoms (PTSD and depression) could have been reduced compared to those not receiving this type of social support.

Another limitation was that the distress symptoms could have been influenced by non-assessed factors such as past victimization, severity of victimization (i.e., across different types of abuse-nonviolent, physical, sexual), and prior mental health history. Future studies should conduct moderation analyses to see whether these variables mitigate the severity of distress symptoms for intimate partner victims of psychopaths. Finally, most participants were former-intimate partners compared to those who reported current involvement. This larger proportion of ex-intimate partners could have impacted the severity of mental and physical health symptoms. However, mitigating this potential problem, no significant differences were found in psychological distress symptoms across current and former intimate partners of psychopaths.

### Future Directions

Few studies have examined the experiences of victims of individuals with psychopathic traits and even fewer have focused on their intimate partners. Therefore, a large part of this study was exploratory. Several recommendations for future studies have been identified, including obtaining a sample of IPV victims whose partners score low in psychopathy, recruiting participants from a broader range of sources, and obtaining more information about prior victimization and prior mental health history. Additionally, future studies should look at gender to see if male and female intimate partners differ in their victimization experiences or psychological distress symptom severity. Moreover, examining the personality traits of victims could provide insight into whether certain personality profiles are more vulnerable to the effects of the psychopath’s victimizing actions. Finally, although many of the consequences reported by the intimate partners were negative, some responses implied resiliency and positive effects, like becoming a stronger person and learning from the experience. These positive experiences are consistent with post-traumatic growth and this, along with protective factors, could be a fruitful area for future research.

### Implications and Conclusions

The current study examined the negative symptomatology experienced by the intimate partners of individuals with psychopathic traits and the results strongly suggest that psychopathy has a wide range of effects for intimate partners—be it emotional, biological, behavioral, cognitive, or interpersonal. Additionally, coping strategies appear to play a very important role in the manifestation of psychological distress symptoms. Understanding the physical and mental health symptoms experienced by intimate partners and the factors that can impact the severity of these symptoms (i.e., level of psychopathic features present, coping) allows for the development of targeted treatment interventions. Specifically, the incidence of PTSD and depression in victims could be reduced by encouraging the use of adaptive coping and by changing maladaptive coping behaviors. By using these strategies, victims of intimate relationships with individuals with psychopathic traits can begin to recover and lead fulfilling lives. Victim centric studies like the current one also provide more insight into community-based psychopathy, particularly in the areas of treatment and prevention. Not much is known about these “successful psychopaths” and by studying their relationship dynamics and behaviors through the eyes of their intimate partners, more effective therapeutic interventions can be developed.

Taken together, the results of the current study indicate that intimate partner victims of psychopaths experience a great deal of physical and mental health consequences that parallel the symptoms reported by victims of general crime, bullying, workplace bullying, and intimate partner violence victimization. The results provide further evidence for the existence of an association between psychopathy and the ensuing psychological distress (PTSD and depression) present in intimate partners. Finally, the results indicate that adaptive and maladaptive coping are related to the severity of these psychological distress symptoms, although the type of abuse itself does not appear to predict depression symptoms.
